# Photosynthetic responses of hydroponically grown basil (*Ocimum basilicum* L.) to drought and high-EC stress

**DOI:** 10.7717/peerj.20728

**Published:** 2026-02-10

**Authors:** Małgorzata Mirgos, Piotr Dąbrowski, Hazem Mohamed Kalaji, Jacek Wróbel, Janina Gajc-Wolska, Bogumiła Pawluśkiewicz, Małgorzata Kunka, Katarzyna Kowalczyk

**Affiliations:** 1 Department of Vegetable and Medicinal Plants, Institute of Horticultural Sciences, Warsaw University of Life Sciences, Warsaw, Poland; 2 Department of Environmental Development and Remote Sensing, Institute of Environmental Engineering, Warsaw University of Life Sciences—SGGW, Warsaw, Poland; 3 Department of Botany and Plant Physiology, Institute of Biology, Warsaw University of Life Sciences, Warsaw, Poland; 4 Department of Bioengineering, West Pomeranian University of Technology in Szczecin, Szczecin, Poland; 5 The National Institute of Horticultural Research, Skierniewice, Poland

**Keywords:** Abiotic stresses, Chlorophyll a fluorescence, JIP-test, Plant traits, Photosynthesis, Basil, Photosynthetic performance, Pulse-modulated amplitude fluorescence, Hydroponic systems

## Abstract

**Background:**

Basil (*Ocimum basilicum* L.), a widely cultivated culinary and medicinal herb in the *Lamiaceae* family, is particularly vulnerable to various environmental stressors. This study examines how water deficit and elevated nutrient-solution electrical conductivity (EC) affect the photosynthetic efficiency of basil plants grown in an nutrient film technique (NFT) hydroponic system.

**Methods:**

Chlorophyll fluorescence was assessed using both continuous-excitation and modulated pulse-amplitude-modulated (PAM) techniques. Fluorescence parameters were monitored in plants at two developmental stages, immature and mature, under drought and high–electrical-conductivity (EC) stress.

**Results:**

Both stressors altered Photosystem II (PSII)—related fluorescence parameters, but high EC stress caused a wider spectrum of changes. In mature plants, those alterations were less pronounced, indicating enhanced tolerance likely due to more efficient electron transport and greater structural stability of the photosynthetic apparatus. The obtained results supported our hypothesis, that drought and high-EC stress would differentially impair photosynthetic efficiency, with drought imposing stronger osmotic limitations on photochemistry and high EC introducing additional ionic constraints. These stresses generated distinct physiological response patterns detectable by chlorophyll fluorescence measurements.

## Introduction

Basil (*Ocimum basilicum* L.) is one of the most widely cultivated members of the *Lamiaceae* family, prized for its aromatic and medicinal qualities (Makri & Kintzios, 2008; Azizah et al., 2023). Beyond culinary uses, basil essencial oils enhance fragrance formulations and prolong the shelf life of food products (Purushothaman et al., 2018; Javid et al., 2024). Historically, it has been employed to alleviate headaches, lower cholesterol, regulate blood sugar and blood pressure, and support kidney function (Azizah et al., 2023; Ahmed, 2016). The herb’s bioactivity derives from its rich profile of essential oils (≈1.5%), phenolic compounds, flavonoids, glycosides, and organic acids (Dzida, 2010; Shahrajabian, Sun & Cheng, 2020; Romano et al., 2022; Du et al., 2023).

Under optimal conditions (23–30 °C), basil thrives, but adequate irrigation is critical for maintaining yield and quality (Walters & Currey, 2019). It is cultivated commercially using various systems, including hydroponics and aquaponics (Avdouli et al., 2021; Maurer et al., 2023; Mourantian et al., 2023; Signorini et al., 2025). Hydroponics minimizes water and nutrient losses, and the total amount of available ions can be determined by monitoring electrical conductivity (EC). An increase or decrease in EC, especially when it exceeds the tolerance level of the plants, can cause nutrient stress (*i.e.,* excess or deficiency), which inhibits growth and development (Hosseini et al., 2021). For basil plants, a salinity level of 5 dS m^−1^ is considered the upper limit of stress tolerance (Avdouli et al., 2021).

Salinity stress disrupts plant osmotic balance in a manner that depends on the salinity source, concentration, and exposure duration. Excess ions in the rhizosphere impose osmotic and ionic stress, disrupting metabolic homeostasis and reducing plant productivity (Zhao et al., 2020). High nutrients concentrations elicit a suite of physiological and morphological responses, including decreased water absorption, stomatal closure, and inhibited photosynthesis, that together diminish growth and yield (Zhu, 2001; Tester & Davenport, 2003).

Basil is considered a moderately salt-tolerant species (Barbieri et al., 2012; Scagel, Bryla & Lee, 2017), although significant intraspecific differences have been reported (Meftahizadeh, Shahhoseini & Ghanbari, 2025; Cruz et al., 2020). Moreover, moderate salinity can stimulate basil’s secondary metabolism, increasing the production of desirable aromatic compounds for both industry and consumers (Scagel, Lee & Mitchell, 2019; Ciriello et al., 2023). However, as Jadczak et al. (2022) point out, any economic assessment of basil cultivation under saline conditions must balance this enhanced phytochemical yield against reductions in fresh biomass, since lower overall plant mass may negate per-area gains in secondary metabolite production.

Although hydroponic systems allow precise control of the nutrient supply tailored to basil’s needs, interruptions to access to the nutrient solution can result in drought and cause a variety of physiological and biochemical changes. Basil plants also demonstrate high variability in their tolerance to water stress (Rahimi et al., 2023). One of the first signs of water deficiency in basil is a decrease in turgor pressure, which leads to inhibition of cell growth and development, especially in stems and leaves (Sirousmehr, Arbabi & Asgharipour, 2014; Barickman et al., 2021; Damalas, 2019). Basil responds to water stress by activating osmoregulatory mechanisms, such as the accumulation of ions, proline and defence compounds. These mechanisms help to maintain water homeostasis and protect against cell damage. Severe dehydration may be partly responsible for the reduction in the fresh weight of basil leaves, although in response to stress, the plant increases the number of secondary metabolites such as phenols, flavonoids. In turn, the carotenoid content may decrease (Al-Huqail et al., 2020).

Basil responds to stress factors at multiple levels, involving physiological, biochemical and morphological processes. Understanding these mechanisms is essential for optimising growing conditions, especially in hydroponic systems, where sudden changes in water availability or nutrient concentration can occur.

Physiological changes, such as declines in photosynthetic performance, manifest before visible morphological symptoms in stressed plants (Al-Huqail et al., 2020; Peng et al., 2022). Because photosynthesis is uniquely sensitive to environmental perturbations, chlorophyll fluorescence kinetics have become a powerful, non-invasive method for quantifying stress impacts on photochemical efficiency. By analysing fluorescence induction curves, researchers can dissect the function of individual electron-transport components, with particular focus on Photosystem II (PSII). As the most stress-vulnerable site in the photosynthetic apparatus, PSII is both an early indicator of environmental damage and a central player in the plant’s adaptive response (Kan et al., 2017; Baker & Rosenqvist, 2004).

Two main approaches are used to measure chlorophyll fluorescence: continuous-excitation fluorescence and pulse-amplitude-modulated (PAM) fluorescence. PAM fluorescence provides information on the actual photosynthetic efficiency of plants, while continuous-excitation fluorescence gauges the potential efficiency of the photosynthetic apparatus. Among the analytical tools applied to continuous-excitation data, the JIP-test is especially valuable. Grounded in the theory of energy fluxes within thylakoid membranes, the JIP-test evaluates Photosystem II (PSII) performance and its response to environmental factors. By linking biophysical aspects of photosynthesis to specific fluorescence parameters, it delivers a detailed assessment of PSII functionality under stress (Kalaji et al., 2017; Strasser, Tsimilli-Michael & Srivastava, 2004). Both methods are suitable for field applications and assessing plant responses under natural conditions, and they complement each other effectively (Baker & Rosenqvist, 2004).

Although numerous studies have examined the effects of different stressors on photosynthetic performance of basil plants (Lazarević et al., 2021; Sperdouli et al., 2024; Oprea et al., 2025), the literature addressing this issue specifically under drought stress and high-EC nutrient solutions remains scarce. Therefore, the primary objective of this study was to enhance our understanding of how the photosynthetic apparatus responds to these two stresses in basil by employing both continuous-excitation and PAM fluorescence techniques. We hypothesized that drought and high-EC stress would differentially impair photosynthetic efficiency, with drought imposing stronger osmotic limitations on photochemistry and high EC introducing additional ionic constraints. Consequently, we expected these stresses to generate distinct physiological response patterns detectable with both measurement approaches.

## Materials & Methods

### Plant growth conditions and experiment plan

The study was carried out in the greenhouse facilities of the Warsaw University of Life Sciences, using *Ocimum basilicum* L. ‘Keira’ (Genovese type), renowned for its medium-sized, dark green leaves.

#### Seedling establishment and early growth

Basil seeds (20 seeds, \∼0.03 g per cube) were sown on 22 August 2023 and 5 September 2023 into mineral-wool cubes (40  × 40  × 75 mm). The first sowing yielded plants for the mature-stage trials, while the second provided juvenile-stage material. All seeds germinated under light and were irrigated with a starter solution (EC 2.0 mS × cm^−1^; pH 5.5) containing (mg × dm^−^^3^): N 170, P 35, K 200, Mg 45, Ca 180, Fe 2, Mn 0.6, B 0.3, Cu 0.15, Zn 0.3, Mo 0.05.

#### Transfer to NFT system

Fourteen days after sowing, seedlings were transferred, one per 100 mm perforated pot (0.4 dm^3^), and spaced at 30 cm intervals in nutrient film technique (NFT) channels. From this point on, plants received a working nutrient solution (EC 2.8 mS × cm^−1^; pH 5.5), whose composition (mg dm^−^^3^) was: N 200, P 50, K 240, Mg 45, Ca 190, Fe 2, Mn 0.6, B 0.3, Cu 0.15, Zn 0.3, Mo 0.05. Solutions were replaced weekly, with daily checks of pH and EC. Greenhouse conditions averaged 24.4–25.4 °C, \∼486 J × cm^−^^2^ × day^−^^1^ solar radiation, ≥50% relative humidity, and 800 ppm CO_2_.

#### NFT infrastructure

Three identical NFT systems (NFT 1–3) each comprised four 7 m channels (1% slope), a lower reservoir, a recirculating pump, and a mesh filter. Nutrient solution was delivered *via* capillary tubing to the channel inlets, flowed as a thin film over the roots, and returned to the reservoir by gravity. A computer-controlled pump maintained continuous circulation. Each system accommodated two groups, 40 mature-phase and 40 immature-phase pots, across its four gutters. For each treatment, four repetitions of 10 pots with basil plants were used.

#### Stress treatments

Stress treatments began at defined ages: for juvenile (immature) plants 14 days after planting (14 DAP); for mature plants 30 days after planting (30 DAP).

1. Control (NFT 1): continuous feeding with working solution.
2. Drought stress (NFT 2): complete withholding of nutrient solution and water for 3 days, starting at 14 DAP (juvenile) or 30 DAP (mature).
3. High-EC stress (NFT 3): provision of a twice-concentrated solution (EC ≈ 8 mS × cm^−1^; pH 5.5) for 3 days. Daily EC and pH measurements were taken with a DGT Mesur Hand instrument (Senmatic, Denmark).

All measurements of plant responses followed the 3-day stress period (Table 1).

**Table 1:** Assessed elements: plant age, onset of stress conditions and type of stress conditions.

Plants age	Start of stress	Stress conditions
Juvenile	14 DAP	Control (NFT 1)
		Drought (NFT 2)
		EC (NFT 3)
Mature	30 DAP	Control (NFT 1)
		Drought (NFT 2)
		EC (NFT 3)

### Measurements and analyses

On the third and final day of stress treatment, three uniformly developed plants from each replicate were selected for measurement of leaf chlorophyll content soil plant analysis development (SPAD) and chlorophyll fluorescence.

#### Leaf chlorophyll content

Leaf chlorophyll content (expressed as SPAD units) was estimated with the portable equipment SPAD-502 (Minolta, Tokyo, Japan). For this purpose, the results of three individual measurements on a fully developed leaf of basil plants growing in a single pot were averaged, and three replicates of such measurements were performed on plants from three individual pots in each combination.

#### Chlorophyll fluorescence measurements

The measurements of chlorophyll *a* fluorescence were performed by using two fluorimeters:

- HandyPEA (Hansatech Instruments Ltd., Pentney, Norfolk, UK),
- TOMI-2 (Photon Systems Instruments, spol. s r.o., Drásov, Czech Republik).

Chlorophyll fluorescence parameters measured by both above mentioned techniques are presented in Table 2.

**Table 2:** Glossary, definition of terms, and formulae used by the chlorophyll a fluorescence parameters (Tsimilli-Michael, 2020, modified).

**Continuous-excitation fluorescence (JIP-test) parameters**	
t_Fm_	Time (in ms) to reach the maximal fluorescence FP (meaningful only when *F*_P_ = *F*_M_)
Area	Total complementary area between the fluorescence induction curve and F = FP (meaningful only when *F*_P_ = *F*_M_)
F_0_≅ F50µs or ≅ F20µs	Fluorescence when all PSII RCs are open (≅ to the minimal reliable recorded fluorescence)
*F*_M_	Maximal fluorescence, when all PSII RCs are closed
*F*_V_≡*F*_M_ –F_0_	Maximal variable fluorescence
*F*_V_/*F*_M_	Maximum quantum yield for primary photochemistry
ABS/RC = M_0_× (1/V_J_) × (1/*ϕ*_Po_)	Absorption flux (exciting PSII antenna Chl *a* molecules) per RC (also used as a unit-less measure of PSII apparent antenna size)
TR_0_/RC = M_0_× (1/V_J_)	Trapped energy flux (leading to Q_A_ reduction), per RC
RE_o_/RC = M_o_× (1/V_J_) × (1 − V_I_)	Electron flux reducing end electron acceptors at the PSI acceptor side, per RC
ET_0_/RC = M_0_× (1/V_J_) × (1 − V_J_)	Electron transport flux (further than Q_A_^−^), per RC
DI_0_/RC = (ABS/RC) − (TR_0_/RC)	Energy flux not intercepted by an RC, dissipated in the form of heat, fluorescence, or transfer to other systems, at time *t* = 0.
*ϕ*_Po_≡ TR_0_/ABS = [1 − (F_0_/*F*_M_)]	Maximum quantum yield for primary photochemistry
*ϕ*_Eo_≡ ET_0_/ABS = [1 − (F_0_/*F*_M_)] × (1 − V_J_)	Quantum yield for electron transport (ET)
*ϕ*_Ro_≡ RE_M_/ABS = [1 − (F_0_/*F*_M_)] × (1 − V_I_)	Quantum yield for reduction of end electron acceptors at the PSI acceptor side (RE)
*ψ*_Eo_≡ ET_0_/TR_0_ = (1 − V_J_)	Efficiency/probability that an electron moves further than Q_A_^−^
*δ*_Ro_≡ RE_0_/ET_0_ = (1 − V_I_)/(1 − V_J_)	Efficiency/probability with which an electron from the intersystem electron carriers is transferred to reduce end electron acceptors at the PSI acceptor side (RE)
N = S_m_× (M_0_/V_J_)	Turnover number (expresses how many times Q_A_ is reduced in the time interval from 0 to t_Fm_)
S_m_= (Area)/(*F*_M_ –F_0_)	Normalized total area above the OJIP curve
PI_abs_	Performance index for energy conservation from photons absorbed by PSII until the reduction of intersystem electron acceptors
PI_tot_	Total performance index for energy conservation from photons absorbed by PSII until the reduction of PSI end electron acceptors
PI_inst._	Instrument-specific parameter
D*F*_abs=_log(PI_abs_)	PSII-relative driving force index on an absorption basis
D*F*_tot_= log(PI_tot_)	Total PSII-relative driving force index
**Pulse-Amplitude-Modulated (PAM) fluorescence parameters**
*F*_p_	Peak fluorescence
*F*_M_’	Maximum fluorescence intensity in a light-exposed sample
*F*_t_	Steady-State fluorescence
F_0_’	The minimum fluorescence intensity in a light-adapted state
Yield = (*F*_M_’ –F)/*F*_M_’	The efficient quantum yield of PSII photochemistry or PSII quantum efficiency represents the fraction of the light energy absorbed by PSI, which drives photosynthetic electron transport
qP = (*F*_M_’ –Ft)/(*F*_M_’ –F_0_)	The photochemical quenching of variable chlorophyll fluorescence
qN = 1 - Fv’ / Fv	The non-photochemical quenching of variable chlorophyll fluorescence
NPQ = (*F*_M_ –*F*_M_′)/*F*_M_′	The non-photochemical quenching of maximum chlorophyll fluorescence
Rfd	Relative Fluorescence Decline

Measurements performed by HandyPEA fluorimeter were conducted on previously marked leaves. The mid-section of the leaf was dark-adapted for at least 25 min before measurements using special leaf clips. A short light flash was applied to the leaf to adjust the detector “gain” level just before measuring the fluorescence transients, and each leaf sample was illuminated with continuous saturating actinic light (3,500 µmol photons m^−2^ s^−1^).

The measurements of PAM performed by TOMI-2 fluorimeter (Photon Systems Instruments, spol. s r.o., Drásov, Czech Republic) were performed on whole plants after continuous-excitation chlorophyll fluorescence. The following protocol was used for PAM measurements:

1. Plants were adapted to darkness for about 20 min in darkroom,
2. First pulse light was activated for 1 s (F_0_ and *F*_M_ measured),
3. Wait until the signal get steady state,
4. Actinic light were activated (*F*_p_ measured),
5. Wait until signal get steady state (*F*_t_ was measured),
6. Next pulses light were activated for 1 s (*F*_m′_ measured).

#### Fresh and dry plant mass

After biometric measurements, plants from the stress conditions and control plants were assessed for fresh herb mass. For this measurement, shoots with leaves were cut above the surface of the substrate from three pots in each of four replicates and weighed on a laboratory balance (RADWAG WTC 2000, Radom, Poland) with an accuracy of 0.01 g. Results are given in g fresh mass (FM) per pot. Dry mass (DM) was determined in percentage at 105 °C by the use of SUP-65W WAMED oven, Poland.

#### Statistical analysis

All chlorophyll *a* fluorescence and weight parameters were statistically analyzed using a two-way analysis of variance (ANOVA) test, where the first factor was the age of the leaves and the second was the stress treatment. The Fisher’s least significant differences test was used as a *post hoc* test at a 0.05 confidence level. The relationships between physiological parameters and plant weight under stress conditions were determined using principal component analysis (PCA) and correlation analysis. Statistica 13.0 program (TIBCO Software Inc., USA) was used to perform the statistical analysis.

## Results

**Figure 1:**
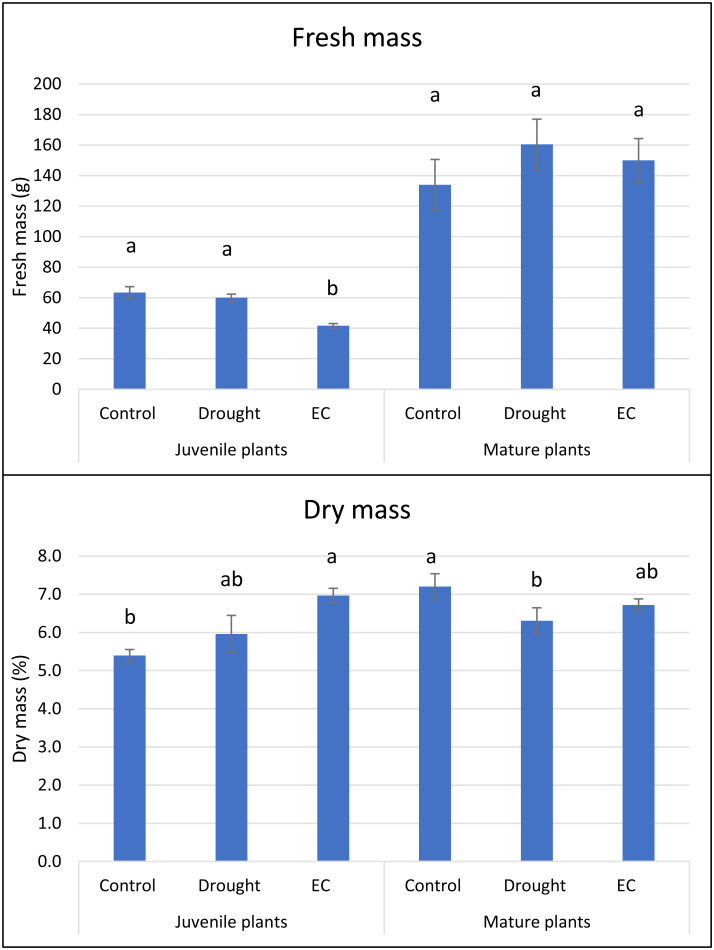
Fresh mass (g) and dry mass (%) of juvenile and mature basil under drought and EC stress conditions. Means values ± standard deviation (SD). Values in each stress factor and age marked by this same letter do not differ significantly (*p* < 0.05).

Drought stress did not significantly reduce fresh mass relative to the control (Fig. 1). In contrast, elevated-EC stress caused a marked decrease in fresh mass compared to both control plants and those under drought. This pattern held for both juvenile and mature plants.

**Figure 2:**
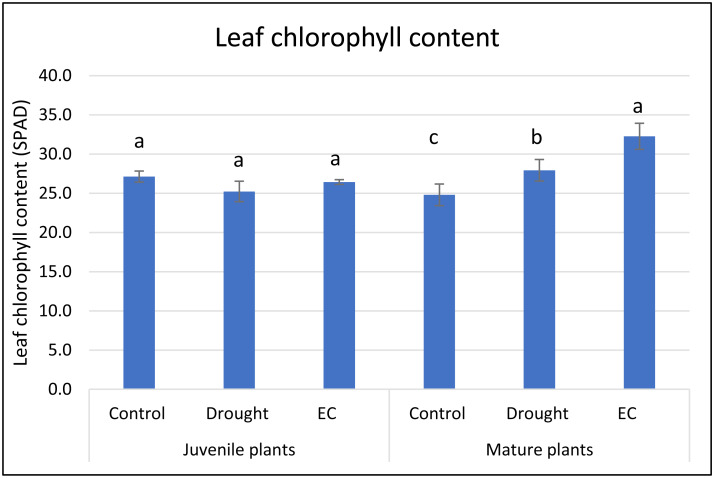
Leaf chlorophyll content (SPAD units) of basil under drought and EC stress condition. Means values ± SD. Values in each stress factor and age marked by this same letter do not differ significantly (*p* < 0.05).

In juvenile plants, EC stress caused an increase in dry mass, whereas in mature plants, a significant decrease in this parameter was observed under drought stress. Leaf chlorophyll content (SPAD units) in juvenile plants was unaffected by either stress (Fig. 2). However, mature plants exhibited a significant SPAD increase under both stresses, with the greatest elevation observed under high-EC conditions.

**Figure 3:**
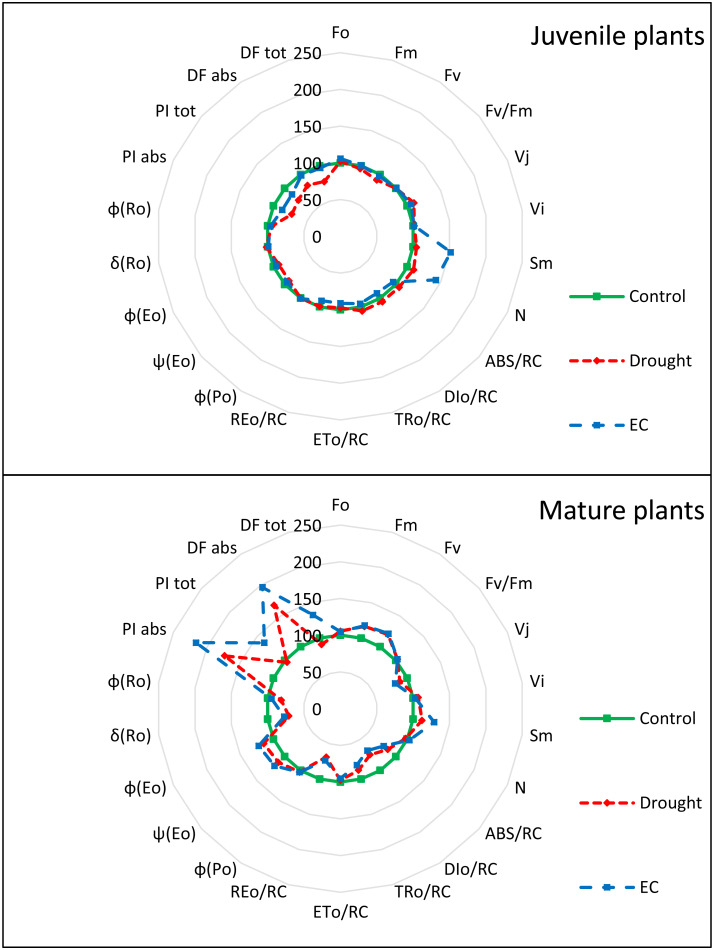
JIP-test parameters of juvenile and mature basil under drought and EC stress conditions, normalized to the values of control plants as radar plots.

Changes in the values of individual parameters of the JIP test depended on the type of stress and the age of the plant (Fig. 3, Table 3). In juvenile plants, the D*F*_abs_ and D*F*_tot_ parameters were the only ones that showed significant changes under drought stress compared to the control. More parameters were affected by EC stress, including F_0_, *F*_V_, S_m_, and N. The largest group consisted of parameters that changed under the influence of both stress factors: V_j_, ET_0_/RC, RE_0_/RC, Ψ_Po_, *ϕ*_Eo_, *δ*_Ro_, PI_abs_, and PI_tot_. Parameters that remained unchanged under both stress conditions included *F*_M_, *F*_V_/*F*_M_, ABS/RC, DI_0_/RC, TR_0_/RC, *ϕ*_Po_, and *ϕ*_Ro_. In mature plants, significantly fewer parameters exhibited significant changes under both stress conditions (ET_0_/RC and N). The S_m_ and PI_tot_ parameters changed only under EC stress, while the only parameter affected solely by drought stress was *δ*_Ro_. No significant differences were found in the values of other parameters measured in control plants and those subjected to stress factors.

**Table 3:** J IP-test parameters of juvenile and mature basil under drought and EC stress conditions. Means values ± SD. Values in each stress factor and age marked by this same letter do not differ significantly (*p* < 0.05).

Age	Juvenile			Mature		
Treatment	Control	Drought	EC	Control	Drought	EC
F_0_	5,109b ± 605	5,211b ± 394	5,407a ± 160	5,384b ± 253	5,677a ± 182	5,642a ± 255
*F*_M_	31,850a ± 2,926	30,550a ± 2,463	32,018a ± 1,859	29,589b ± 2,227	34,496a ± 3,166	34,888a ± 1,760
*F*_V_	27,407a ± 1,393	25,006b ± 1,664	26,711ab ± 1,602	24,205b ± 2,012	28,986a ± 2,838	29,329a ± 1,505
*F*_V_/*F*_M_	0.829a ± 0.019	0.829a ± 0.007	0.834a ± 0.003	0.818b ± 0.007	0.840a ± 0.008	0.841a ± 0.005
V_j_	0.43b ± 0.05	0.47a ± 0.04	0.46a ± 0.02	0.51a ± 0.03	0.46b ± 0.04	0.42c ± 0.02
V_i_	0.78a ± 0.02	0.79a ± 0.02	0.79a ± 0.02	0.75c ± 0.01	0.80a ± 0.04	0.77b ± 0.02
S_m_	20.85b ± 2.23	21.83b ± 2.72	31.62a ± 2.19	19.66b ± 2.02	22.07b ± 2.57	25.35a ± 1.56
N	29.94b ± 4.57	32.77b ± 2.52	42.78a ± 3.84	30.31a ± 2.28	29.61a ± 1.94	31.31a ± 1.90
ABS/RC	1.73a ± 0.10	1.83a ± 0.19	1.64a ± 0.08	1.90a ± 0.16	1.61b ± 0.15	1.47c ± 0.13
DI_0_/RC	0.296a ± 0.037	0.313a ± 0.039	0.272a ± 0.014	0.346a ± 0.040	0.258b ± 0.029	0.235c ± 0.023
TR_0_/RC	1.43a ± 0.09	1.51a ± 0.15	1.36a ± 0.07	1.54a ± 0.12	1.35b ± 0.12	1.24c ± 0.11
ET_0_/RC	0.814a ± 0.09	0.794b ± 0.08	0.743b ± 0.03	0.753a ± 0.05	0.734a ± 0.05	0.717a ± 0.06
RE_0_/RC	0.320a ± 0.04	0.318b ± 0.01	0.292c ± 0.02	0.395a ± 0.03	0.271b ± 0.04	0.290b ± 0.02
*φ*_Po_	0.829a ± 0.02	0.829a ± 0.007	0.834a ± 0.003	0.818b ± 0.007	0.840a ± 0.008	0.841a ± 0.005
*ψ*_Eo_	0.568a ± 0.051	0.526b ± 0.041	0.544b ± 0.016	0.488c ± 0.031	0.545b ± 0.036	0.579a ± 0.022
*φ*_Eo_	0.472a ± 0.051	0.436b ± 0.032	0.453b ± 0.014	0.399c ± 0.028	0.458b ± 0.033	0.487a ± 0.019
*δ*_Ro_	0.397b ± 0.057	0.403a ± 0.040	0.394b ± 0.030	0.525a ± 0.035	0.372b ± 0.068	0.405b ± 0.025
*φ*_Ro_	0.185a ± 0.057	0.176a ± 0.040	0.179a ± 0.030	0.208a ± 0.035	0.170b ± 0.068	0.197a ± 0.025
PI_abs_	3.95a ± 0.45	2.87b ± 0.45	3.43c ± 0.28	2.32c ± 0.60	4.02b ± 0.90	5.01a ± 0.81
PI_tot_	2.67a ± 0.42	2.01b ± 0.54	2.33b ± 0.39	2.52b ± 0.40	2.43b ± 0.81	3.45a ± 0.82
D*F*_abs_	0.57a ± 0.15	0.47b ± 0.10	0.56a ± 0.05	0.35c ± 0.10	0.59b ± 0.11	0.69a ± 0.07
D*F*_tot_	0.39a ± 0.11	0.30b ± 0.14	0.38a ± 0.08	0.40b ± 0.07	0.36b ± 0.16	0.53a ± 0.10

**Figure 4:**
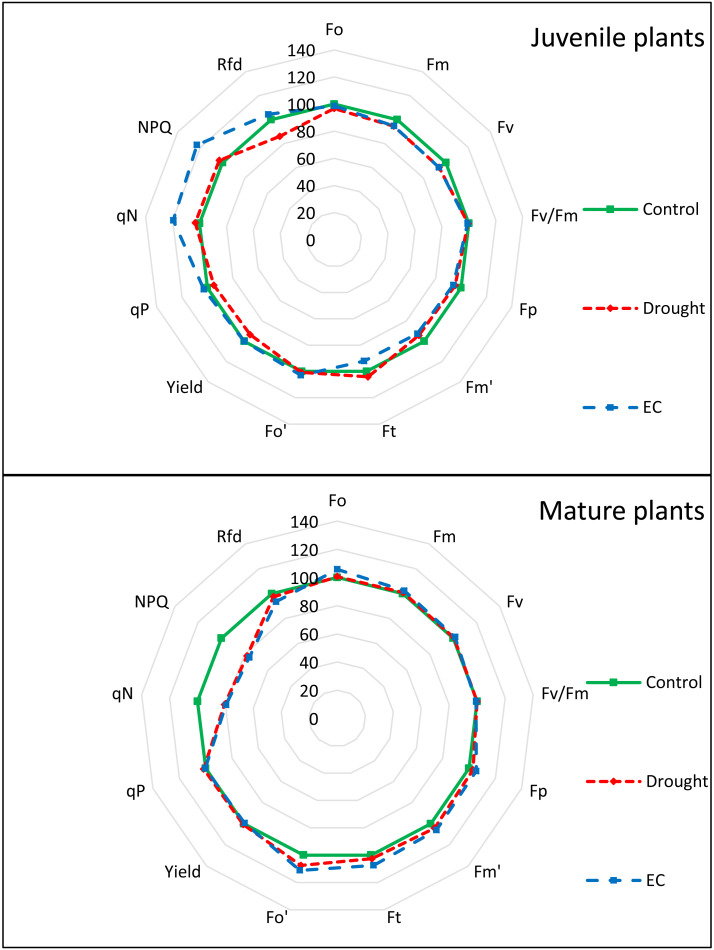
PAM parameters of juvenile and mature basil under drought and EC stress conditions, normalized to the values of control plants as radar plots.

**Figure 5:**
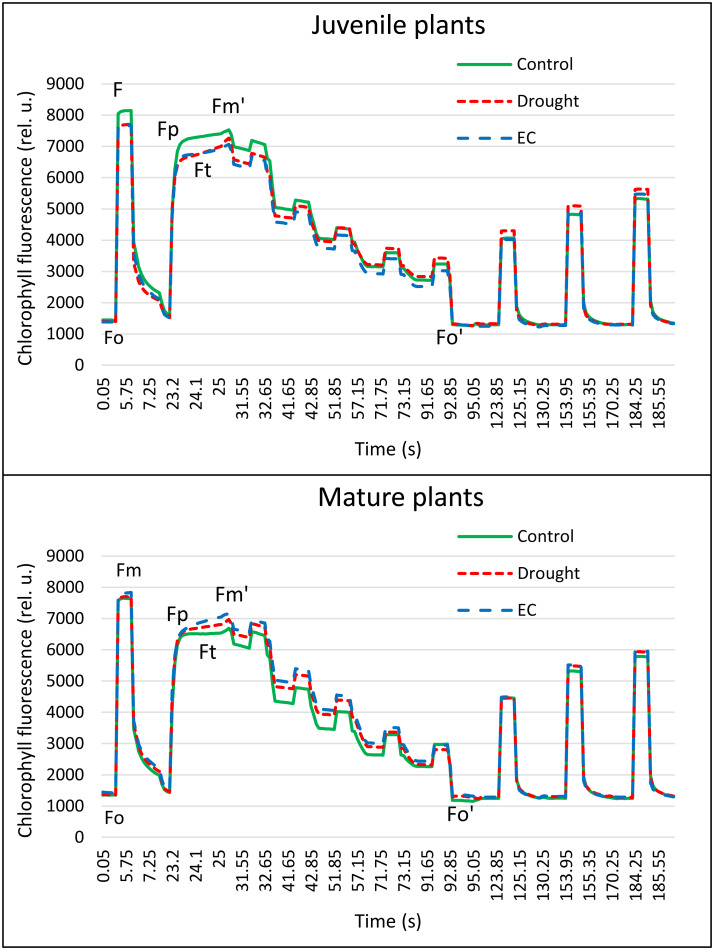
PAM curves of chlorophyll *a* fluorescence of juvenile and mature basil under drought and EC stress conditions.

Similarly, changes in the values of individual PAM parameters depended on the type of stress and plant age (Figs. 4 and 5, Table 4). In juvenile plants, *F*_M_’ was the only parameter sensitive to both stress factors. Yield, qP, and Rfd were sensitive only to drought stress, while *F*_t_, qN, and non-photochemical quenching (NPQ) were sensitive only to EC stress. Among the PAM parameters measured in mature plants, only F_0_’ was affected by both stress factors. No significant differences were found in the values of the other parameters measured in control plants and those subjected to stress factors. Changes at specific points of the PAM chlorophyll fluorescence curve and the overall course of the curve also provide valuable information on photosynthetic efficiency and environmental stress in plants, allowing for a better understanding of their responses to stress conditions depending on age (Fig. 5).

**Table 4:** PAM parameters of juvenile and mature basil under drought and EC stress conditions. Means values ± SD. Values in each stress factor and age marked by this same letter do not differ significantly (*p* < 0.05).

Age	Juvenile			Mature		
Treatment	Control	Drought	EC	Control	Drought	EC
F_0_	1,443a ± 78	1,398a ± 39	1,425a ± 122	1,352a ± 105	1,361a ± 99	1,431a ± 81
*F*_M_	8,144a ± 359	7,699a ± 206	7,718a ± 563	7,644a ± 384	7,699a ± 231	7,823a ± 265
*F*_V_	6,701a ± 334	6,302a ± 193	6,293a ± 493	6,293a ± 313	6,338a ± 242	6,393a ± 192
*F*_V_/*F*_M_	0.823a ± 0.009	0.818a ± 0.005	0.815a ± 0.012	0.823a ± 0.009	0.823a ± 0.013	0.817a ± 0.005
*F*_p_	7,905a ± 381	7,535a ± 151	7,428a ± 599	7,135a ± 424	7,353a ± 233	7,527a ± 231
*F*_M_’	7,107a ± 274	6,694b ± 166	6,575b ± 787	6,495a ± 470	6,761a ± 209	6,889a ± 191
*F*_t_	2,723a ± 79	2,837a ± 75	2,507b ± 186	2,264a ± 149	2,324a ± 133	2,434a ± 192
F_0_’	1,273a ± 47	1,282a ± 28	1,309a ± 3	1,206b ± 57	1,299a ± 32	1,340a ± 94
Yield	0.617a ± 0.012	0.576b ± 0.006	0.617a ± 0.022	0.651a ± 0.011	0.656a ± 0.020	0.647a ± 0.021
qP	0.751a ± 0.013	0.713b ± 0.007	0.773a ± 0.009	0.801a ± 0.021	0.812a ± 0.027	0.803a ± 0.019
qN	0.155b ± 0.015	0.160b ± 0.004	0.185a ± 0.054	0.183a ± 0.023	0.148a ± 0.025	0.146a ± 0.018
NPQ	0.146b ± 0.015	0.150b ± 0.005	0.179a ± 0.063	0.178a ± 0.027	0.139a ± 0.026	0.136a ± 0.018
Rfd	1.99a ± 0.09	1.71b ± 0.05	2.08a ± 0.05	2.38a ± 0.12	2.32a ± 0.18	2.23a ± 0.22

It was found that under both stress conditions, there was a relationship between some JIP-test parameters of chlorophyll fluorescence and biometric traits. Fresh mass was positively correlated with V_i_ and PI_abs_ in juvenile plants subjected to drought stress but negatively with RE_0_/RC, *δ*_Ro_, and *ϕ*_Eo_. Dry mass was positively correlated with V_j_, *δ*_Ro_, and *ϕ*_Ro_ but negatively only with PI_abs_. Leaf chlorophyll content was positively correlated with *F*_M_ and *F*_V_, and negatively with S_m_ and N. Fresh mass was positively correlated with *F*_V_/*F*_M_, V_i_, and *ϕ*_Po_ but negatively with ET_0_/RC, RE_0_/RC, and *ϕ*_Ro_ in mature plants subjected to drought stress. Dry mass was correlated only with ET_0_/RC, and this was a positive relationship. Leaf chlorophyll content was significantly and positively correlated with *F*_M_, *F*_V_, *F*_V_/*F*_M_, Sm, *ϕ*_Po_, PI_abs_, and D*F*_abs_, but negatively with ABS/RC, DI_0_/RC, TR_0_/RC, and ET_0_/RC.

Fresh mass was positively correlated with ET_0_/RC and PI_abs_ in juvenile plants subjected to EC stress (Table 5). A negative correlation was confirmed between this parameter and F_0_ and N. Dry mass was positively correlated with F_0_ and S_m_, but negatively with ET_0_/RC and *ϕ*_Po_. Leaf chlorophyll content was positively correlated with *F*_M_ and *F*_V_, and negatively with S_m_ and N. Fresh mass was positively correlated with *F*_V_/*F*_M_, V_i_, and D*F*_abs_ in mature plants subjected to EC stress but negatively with V_j_, ABS/RC, DI_0_/RC, TR_0_/RC, RE_0_/RC, and *δ*_Ro_. Leaf chlorophyll content was significantly and positively correlated with *F*_V_, *F*_V_/*F*_M_, V_i_, S_m_, *ϕ*_Po_, PI_abs_, and D*F*_abs_, but negatively with V_j_, ABS/RC, DI_0_/RC, TR_0_/RC, ET_0_/RC, RE_0_/RC, and *δ*_Ro_.

**Table 5:** The Pearson correlation coefficient (r) between juvenile and mature basil biometric parameters under drought stress conditions and JIP-test parameters. Values marked in bold are statistically significant (*p* < 0.05).

Parameter	Juvenile						Mature					
	Fresh mass (g)		Dry mass (%)		SPAD		Fresh mass (g)		Dry mass (%)		SPAD	
	Drt.	EC	Drt.	EC	Drt.	EC	Drt.	EC	Drt.	EC	Drt.	EC
F_0_	0.21	**−0**.**62**	−0.24	**0**.**66**	−0.38	−0.46	−0.08	0.57	0.27	−0.05	0.24	0.47
*F*_M_	0.33	0.18	−0.59	−0.05	**0**.**64**	0.18	0.44	0.20	−0.33	0.16	**0**.**79**	0.59
*F*_V_	0.40	0.13	−0.57	0.02	**0**.**73**	0.07	0.46	0.27	−0.35	0.09	**0**.**80**	**0**.**65**
*F*_V_/*F*_M_	0.34	−0.43	0.25	0.60	0.40	**−0**.**99**	**0**.**54**	**0**.**66**	−0.43	−0.23	**0**.**83**	**0**.**81**
V_j_	−0.43	−0.51	**0**.**73**	0.51	0.35	−0.23	0.18	**−0**.**66**	0.04	0.25	−0.28	**−0**.**73**
V_i_	**0**.**94**	0.09	−0.63	0.13	0.34	−0.62	**0**.**89**	**0**.**75**	−0.37	−0.19	0.55	**0**.**77**
Sm	−0.47	**−0**.**92**	0.40	**0**.**82**	**−0**.**80**	−0.35	0.28	0.59	−0.53	−0.34	**0**.**74**	**0**.**73**
N	−0.44	**−0**.**72**	0.29	0.62	**−0**.**68**	−0.13	−0.19	−0.35	−0.22	0.05	−0.17	−0.60
ABS/RC	−0.14	0.29	−0.13	−0.31	0.04	0.43	−0.45	**−0**.**70**	0.45	0.29	**−0**.**80**	**−0**.**84**
DI_0_/RC	−0.24	0.40	−0.21	−0.48	−0.17	**0**.**73**	−0.51	**−0**.**70**	0.42	0.25	**−0**.**81**	**−0**.**83**
TR_0_/RC	−0.11	0.25	−0.10	−0.25	0.10	0.33	−0.43	**−0**.**70**	0.45	0.31	**−0**.**80**	**−0**.**84**
ET_0_/RC	0.29	**0**.**75**	**−0**.**70**	**−0**.**76**	−0.27	0.61	**−0**.**74**	−0.67	**0**.**62**	0.37	**−0**.**89**	**−0**.**92**
RE_0_/RC	**−0**.**63**	0.14	0.30	−0.25	−0.12	0.56	**−0**.**72**	**−0**.**78**	0.46	0.28	−0.78	**−0**.**89**
*φ*_Po_	0.35	−0.44	0.24	0.60	0.41	**−0**.**98**	**0**.**55**	0.66	−0.42	−0.21	**0**.**82**	**0**.**81**
*ψ*_Eo_	0.43	0.51	−0.73	−0.51	−0.35	0.23	−0.18	0.66	−0.04	−0.25	0.28	0.73
*φ*_Eo_	0.50	0.45	−0.75	−0.42	−0.32	0.07	−0.06	0.66	−0.11	−0.25	0.41	0.75
*δ*_Ro_	**−0**.**76**	−0.33	**0**.**82**	0.20	0.06	0.24	−0.58	**−0**.**82**	0.30	0.24	−0.59	**−0**.**86**
*φ*_Ro_	**−0**.**92**	−0.17	**0**.**70**	−0.04	−0.28	0.52	**−0**.**90**	−0.67	0.34	0.16	−0.46	−0.65
PI_abs_	**0**.**75**	**0**.**68**	**−0**.**67**	−0.54	0.53	−0.11	0.20	0.60	−0.36	−0.33	**0**.**70**	**0**.**79**
PI_total_	−0.24	0.32	0.15	−0.35	0.21	0.38	−0.26	0.29	−0.25	−0.25	0.55	0.52
D*F*_abs_	0.56	0.12	−0.59	−0.06	−0.24	−0.28	0.23	**0**.**67**	−0.31	−0.27	**0**.**66**	**0**.**80**
D*F*_Total_	−0.30	−0.33	0.35	0.21	−0.34	−0.16	−0.30	0.39	−0.20	−0.25	0.50	0.58

It was also found that under both stress conditions, there was a relationship between some PAM chlorophyll fluorescence parameters and biometric traits (Table 6). Fresh mass was positively correlated with Yield and qP in juvenile plants subjected to drought stress but negatively with qN and NPQ. Dry mass was negatively correlated with Yield, qP and Rfd. Leaf chlorophyll content was positively correlated with F_0_, *F*_M_, and *F*_M_’, Yield, qP, and Rfd. Fresh mass was positively correlated with *F*_0′_ and *F*_M′_ but negatively with qN and NPQ in mature plants subjected to drought stress. Dry mass was positively correlated with *F*_0_ andnegatively with F_0_’. Leaf chlorophyll content was correlated negatively with Yield and Rfd.

**Table 6:** The Pearson correlation coefficient (r) between juvenile and mature basil biometric parameters under EC stress conditions and PAM parameters. Values marked in bold are statistically significant (*p* < 0.05).

Parameter	Juvenile						Mature					
	Fresh mass (g)		Dry mass (%)		SPAD		Fresh mass (g)		Dry mass (%)		SPAD	
	Drt	EC	Drt	EC	Drt	EC	Drt	EC	Drt	EC	Drt	EC
F_0_	0.08	−0.06	−0.41	−0.17	**0**.**80**	0.64	0.33	0.38	**0**.**59**	0.19	−0.34	0.46
*F*_M_	0.30	0.44	−0.38	−0.61	**0**.**72**	0.61	0.08	0.09	0.21	0.18	−0.17	0.12
*F*_V_	0.32	0.52	−0.35	−0.67	0.66	0.57	−0.03	−0.05	0.03	0.16	−0.07	−0.05
*F*_V_/*F*_M_	0.27	**0**.**81**	0.03	**−0**.**75**	−0.02	0.06	−0.33	**−0**.**56**	−0.51	−0.14	0.26	−0.65
*F*_p_	0.29	0.47	−0.43	−0.63	0.63	0.62	0.29	0.35	−0.13	−0.04	0.22	0.37
*F*_M_’	0.49	0.49	−0.47	−0.64	**0**.**69**	0.55	**0**.**57**	0.41	−0.01	0.00	0.10	0.35
*F*_t_	−0.61	0.61	0.52	−0.76	−0.15	0.70	0.23	0.13	−0.25	0.09	0.47	0.20
F_0_’	−0.22	−0.50	0.04	0.39	0.37	0.01	**0**.**77**	−0.21	**−0**.**58**	−0.29	0.46	0.47
Yield	**0**.**72**	0.13	**−0**.**67**	−0.26	**0**.**73**	0.16	0.35	0.31	0.38	−0.20	**−0**.**60**	0.09
qP	**0**.**68**	**−0**.**76**	**−0**.**66**	**0**.**80**	**0**.**75**	**−0**.**68**	0.22	−0.12	0.17	−0.35	−0.41	0.16
qN	**−0**.**78**	−0.51	0.34	0.61	0.25	−0.39	**−0**.**96**	**−0**.**72**	0.35	0.31	−0.44	−0.52
NPQ	**−0**.**77**	−0.48	0.35	0.58	0.26	−0.40	**−0**.**96**	**−0**.**74**	0.31	0.29	−0.42	−0.54
Rfd	0.58	−0.62	**−0**.**62**	0.61	**0**.**79**	−0.44	−0.32	−0.16	0.54	−0.01	**−0**.**82**	−0.20

Fresh mass was positively correlated with *F*_V_/*F*_M_ in juvenile plants subjected to EC stress but negatively with qP. In the case of dry mass, it was precisely the opposite. *F*_V_/*F*_M_ was correlated negatively, and qP was correlated positively with this parameter. Leaf chlorophyll content was negatively correlated only with one parameter –qP. In mature plants subjected to EC stress, only *F*_V_/*F*_M_, qN and NPQ were correlated negatively with fresh mass.

**Figure 6:**
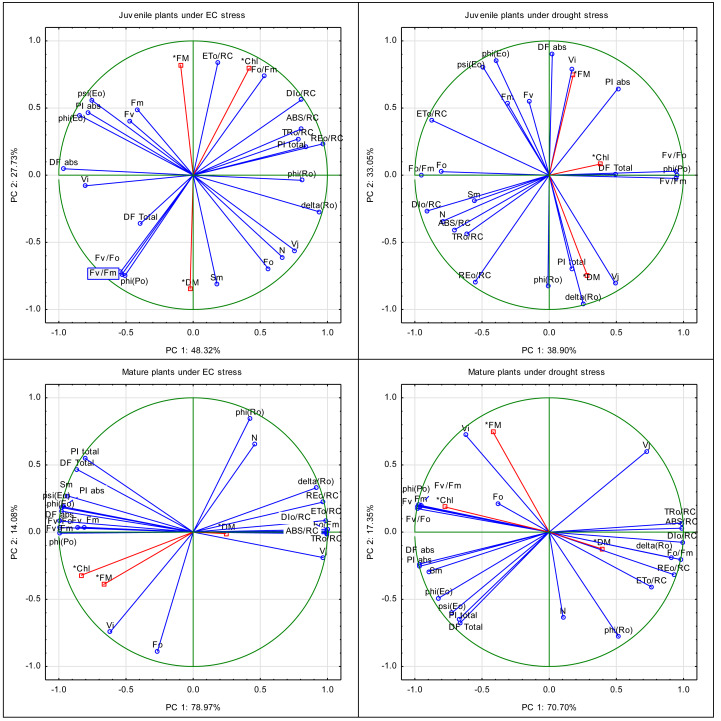
Principal component analysis of the drought and EC effect on the JIP-test parameters, leaf chlorophyll content, and biometric parameters (fresh and dry mass) of juvenile and mature basil. Vector graphs showing the relative “contribution” of each input variable to the formation of the principal components.

The principal components for JIP-test parameters in juvenile plants subjected to EC stress explained 48.32% and 27.73% of the variance, respectively, for PC1 and PC2 (Fig. 6). Parameters associated with primary chlorophyll fluorescence, such as *F*_M_, and *F*_V_, along with F_0_/*F*_M_, V_J_, and V_I_, were strongly correlated and formed a tight cluster on the plot, indicating their coordinated response to this stress. Parameters indicating the electron transport efficiency (TR_0_/RC, ET_0_/RC, *ψ*_Eo_, *δ*_Ro_, *φ*_Ro_) were positioned separately, suggesting their functional independence from the initial fluorescence phase. parameters related to light absorption and energy distribution (ABS/RC, *φ*_Po_, RE_0_/RC) were moderately scattered, possibly reflecting variable sensitivity to EC stress. Dry weight of juvenile plants treated by this stress was correlated with Sm parameter, and SPAD was correlated with ET_0_/RC and F_0_/*F*_M_. The separation of plants along PC2 implies that this component plays a key role in differentiating juvenile plants under EC stress. The relatively high variance explained by PC2 suggests that juvenile plants show a complex and multidimensional response to salinity, particularly through changes in energy distribution and quantum efficiency.

The principal components for JIP-test parameters in mature plants subjected to EC stress explained 78.97% (PC1) and 14.08% (PC2) of the variance, respectively (Fig. 6). Most variables grouped along PC1, indicating a more uniform response in mature plants. JIP-test parameters, such as F_0_, *F*_V_, F_0_/*F*_M_, V_J_, V_I_, and *F*_V_/F_0_, were tightly clustered and showed high interdependence, capturing core PSII activity under this stress. Electron transport-related variables (DI_0_/RC, TR_0_/RC, ET_0_/RC, *ψ*_Eo_, *φ*_Eo_) and performance indices (PI_ABS_, PI_total_) were distinctly located, highlighting their relevance in stress adaptation mechanisms. Light absorption efficiency (ABS/RC) and quantum yield indicators (*φ*_Po_) contributed to the structure of PC1 but were more dispersed. Fresh weight, dry weight, and leaf chlorophyll content occupied different regions, reinforcing their distinct functional response. The dominant role of PC1 suggests that EC stress induces a consistent physiological shift in mature plants, with fewer variable-specific interdependencies. The lower contribution of PC2 implies that the stress response in mature individuals is more homogenous than in juvenile plants.

For juvenile plants exposed to drought stress, PC1 and PC2 explained 38.90% and 33.05% of the total variance, respectively. Parameters such as F_0_, *F*_M_, and *F*_V_, along with F_0_/*F*_M_, V_J_, and S_m_, clustered closely and were located in one region of the PCA plot, reflecting their joint role in early PSII functionality. Electron transport parameters (DI_0_/RC, TR_0_/RC, ET_0_/RC, RE_0_/RC, *φ*_Po_, *φ*_Eo_, *δ*_Ro_, *φ*_Ro_) were grouped in a separate section, indicating their relatively independent response pattern. Notably, the wide dispersion of energy transfer and yield parameters suggests nuanced regulation under water deficit. Fresh weight was correlated with V_I_, D*F*_ABS_ and PI_ABS_ parameters, leaf chlorophyll content (SPAD units) was correlated with D*F*_total_, and dry weight with PI_total_, *φ*_Ro_ and*δ*_Ro._ Differentiation of individuals along PC2 indicates that this component significantly contributes to the variability in drought response in juvenile plants. The high share of variance explained by PC2, more than in EC-stressed juvenile plants, points to a dynamic and diverse adjustment of photosynthetic mechanisms and biomass accumulation under water limitation.

In mature plants subjected to drought stress, PC1 accounted for 70.70% of the variance, while PC2 explained 17.35%. Most JIP-test parameters contributed primarily to PC1, suggesting a well-defined and uniform stress response. Fluorescence parameters (F_0_, *F*_M_, F_0_/*F*_M_, V_J_, V_I_, *F*_V_, *F*_V_/F_0_) were grouped closely, as were transport-related parameters (ABS/RC, DI_0_/RC, TR_0_/RC, ET_0_/RC, RE_0_/RC, *ψ*_Eo_, *φ*_Eo_, *φ*_Ro_), reflecting synchronized shifts in PSII behavior. Performance indices (PI_ABS_, PI_total_) and energy dissipation indicators (D*F*_ABS_, D*F*_total_) contributed strongly to PC1 and showed high coordination with the other variables. The separation of biometric parameters (*fresh weight, dry weight*) suggests that biomass-related changes follow an independent trajectory. However, leaf chlorophyll content was correlated with *φ*_Po_*F*_V_/*F*_M_, *F*_V_/F_0_, *F*_V,_ and *F*_M_ parameters. Due to the dominant explanatory power of PC1, it is likely that mature plants rely on a robust and consolidated physiological strategy to cope with drought. The relatively minor contribution of PC2 highlights the low diversity in the stress response at this stage of development, in contrast to juvenile plants.

**Figure 7:**
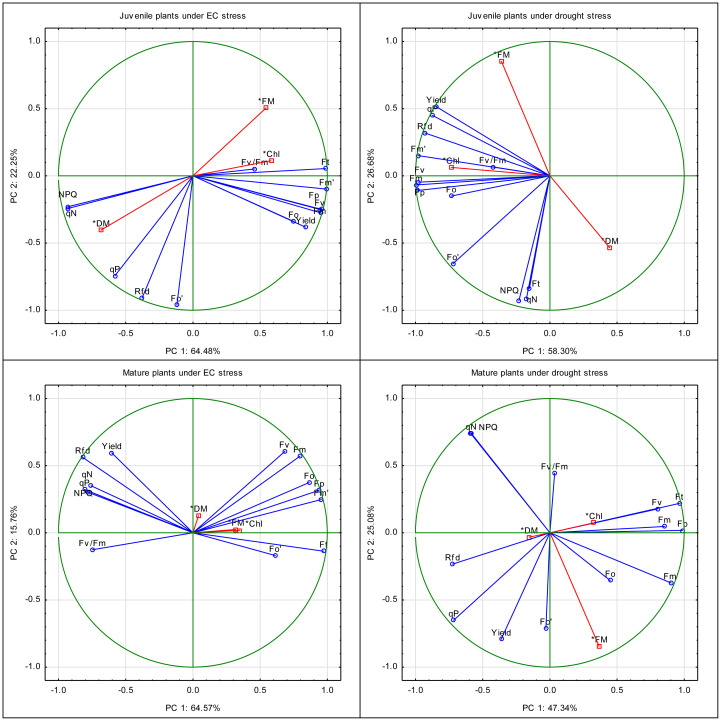
Principal component analysis of the drought and EC effect on the PAM parameters, leaf chlorophyll content, and biometric parameters (fresh and dry mass) of juvenile and mature basil. Vector graphs showing the relative “contribution” of each input variable to the formation of the principal components.

The principal components for PAM parameters and biomass traits in juvenile plants subjected to EC stress explained 64.48% (PC1) and 22.25% (PC2) of the variance (Fig. 7). Parameters associated with PSII maximum fluorescence and efficiency, such as F_0_, *F*_V_, *F*_V_/*F*_M_, *F*_M_’, *F*_t_, and F_0_’, were grouped together and contributed strongly to PC1, suggesting that EC stress affects the fundamental photochemical activity of PSII in juvenile plants. Photochemical quenching (qP) and quantum yield (Yield) also contributed to PC1, which can suggest their correlation with PSII performance. In contrast, parameters associated with non-photochemical quenching (qN, NPQ) and maximum fluorescence in the light-adapted state (*F*_M_, *F*_p_) were more closely associated with PC2, suggesting that this component captures variation in energy dissipation mechanisms. Biometric parameters (fresh weight, dry weight) were clearly separated from chlorophyll fluorescence indicators and showed limited correlation with the primary group of PSII-related traits. However, the leaf chlorophyll content (SPAD units) was correlated with the *F*_V_/*F*_M_ parameter. The differentiation along PC2 suggests that non-photochemical mechanisms and thermal energy dissipation play an important role in early stress adaptation in juvenile plants under salinity.

In mature plants subjected to EC stress, PC1 and PC2 explained 64.57% and 15.76% of the total variance, respectively (Fig. 7). A wide range of fluorescence indicators, including F_0_, *F*_M_, *F*_V_, *F*_M_’, *F*_t_, *F*_V_/*F*_M_, *F*_p_ clustered along PC1. This phenomenon can be interpreted as a high level of correlation and reflects a coordinated impact of EC stress on photochemical efficiency. Photochemical yield (Yield) and quenching (qP) were also associated with PC1, while qN, NPQ, and leaf chlorophyll content contributed mainly to PC2, highlighting their role in differentiating plants according to non-photochemical regulatory responses. On the other hand, fresh and dry weight showed a closer relation to PC1, which suggests that, unlike in juvenile plants, stress effects on biomass in mature individuals are more closely tied to photochemical performance. The dominance of PC1 in explaining variance reflects a consolidated physiological response of mature plants to salinity, with less variation in adaptive traits captured by PC2. This may indicate a more uniform stress response or a more stabilized energy-use strategy in mature plants.

The PCA for juvenile plants under drought stress revealed that PC1 explained 58.30% of the variance, while PC2 accounted for 26.68% (Fig. 7). Such fluorescence parameters as F_0_, *F*_M_, *F*_V_/*F*_M_, *F*_M_’, F_0_’ and quantum yield (Yield) grouped strongly along PC1, indicating their primary role in capturing the photosynthetic effect of drought. Parameters such as *F*_V_, *F*_p_, *F*_t_, and particularly qN and NPQ, contributed mainly to PC2, reflecting variability in non-photochemical quenching and heat dissipation mechanisms under water stress. The clear separation of qN and NPQ from other variables suggests their importance as specific markers of photoprotection and stress regulation in drought conditions. Fresh weight, dry weight, and leaf chlorophyll content were located separately, again indicating limited correlation with fluorescence parameters. The notable variance explained by PC2 suggests that drought triggers complex, multi-pathway responses in juvenile plants, especially in the regulation of excess energy and PSII protection mechanisms.

In mature plants under drought stress, PC1 and PC2 explained 47.34% and 25.08% of the variance, respectively (Fig. 7). Parameters related to maximum and variable fluorescence (F_0_, *F*_M_, *F*_V_, *F*_V_/*F*_M_, *F*_M_’, *F*_t_, F_0_’, *F*_p_) were spread across both PCs, suggesting that drought stress influences multiple facets of PSII function in a non-uniform way in mature plants. Yield and qP aligned with PC1, while NPQ and qN were more strongly related to PC2, showing the role of energy dissipation and heat regulation in distinguishing responses among individuals. Biomass indicators (fresh weight, dry weight, leaf chlorophyll content) were separated from fluorescence variables and did not form a cohesive cluster, implying their distinct response path under drought. Compared to other treatments, mature plants under drought stress were characterized by more dispersed trait distribution across both PCs, with no single dominant cluster. This indicates a heterogeneous stress response that may reflect age-related physiological limitations in photoprotection and resource allocation.

## Discussion

Our findings indicate that drought and high-EC stress impaired basil physiology through distinct mechanisms, consistent with our hypothesis. Although drought did not reduce fresh mass, the decline observed under EC stress suggests that ion toxicity and nutrient imbalances impose stronger limitations on growth than osmotic restriction alone (Chaves, Maroco & Pereira, 2003; Silva et al., 2024; Munns & Tester, 2008; Qiu et al., 2021). The comparable responses of juvenile and mature plants to both stresses suggest that basil’s developmental stage does not substantially alter fundamental tolerance mechanisms, unlike in species where juvenile plants exhibit weaker osmotic adjustment or less-developed root systems (Farooq et al., 2009; Rankenberg et al., 2021).

The increase in dry mass under EC stress, absent under drought, likely reflects salt-induced accumulation of osmolytes and structural solutes that maintain turgor and stabilize proteins, thereby increasing dry matter fraction despite reduced water content (Parida & Das, 2005). Mechanistically, this indicates that salinity triggers a metabolic shift toward the synthesis and storage of compatible solutes, whereas drought primarily causes water-conservation responses rather than extensive osmolyte buildup.

Changes in leaf chlorophyll content, particularly the increase observed in mature plants, may reflect activation of photoprotective pigment systems. Under both stresses but more strongly under EC conditions, enhanced chlorophyll content could support ROS-scavenging capacity and maintain PSII integrity in the presence of ionic perturbations (Baker & Rosenqvist, 2004; Kalaji et al., 2017; Ashraf, Nawazish & Athar, 2007). This suggests that mature plants may upregulate pigment-based protection more effectively than juvenile plants.

Patterns in fluorescence parameters suggest distinct mechanistic limitations under drought *versus* salinity stress. In juvenile plants, drought primarily affected D*F*_abs_ and D*F*_tot_, pointing to restrictions in excitation energy capture and transfer efficiency, consistent with osmotic inhibition of electron transport. In contrast, EC stress altered a broader suite of parameters—including F_0_, *F*_V_, S_m_, and N, which may indicate combined impacts on antenna function, reaction center connectivity, and downstream electron sinks (Munns & Tester, 2008; Kalaji et al., 2016; Liu, Li & Sun, 2011). Greater parameter sensitivity to salinity implies that ionic stress disrupts multiple components of the photosynthetic machinery, not only early photochemical events but also energy distribution and utilization.

In mature plants, fewer parameters changed under both stresses, suggesting higher stability of PSII structure and more effective regulation of electron transport (Siddiqui, Al-Whaibi & Basalah, 2011). The selective sensitivity of S_m_ and PI_tot_ to EC stress, and of *δ*_Ro_ to drought, indicates that mature plants partition their responses, modulating specific segments of the electron transport chain depending on stress type rather than triggering broad systemic adjustments (Munns & Tester, 2008).

Correlations between biomass traits and fluorescence metrics further illustrate divergent stress physiology. In drought-exposed juveniles, positive correlations between fresh mass and Vj and PI_abs_ indicate that maintaining efficient primary photochemistry supports growth under water deficit, while negative relationships involving RE_0_/RC and *ϕ*_Eo_ suggest that drought-induced bottlenecks in electron transport constrain biomass production (Kalaji et al., 2016; Munns, 2002; Dabrowski et al., 2019). Under EC stress, contrasting correlations of dry mass with F_0_, S_m_, and ET_0_/RC highlight the interplay between altered antenna function, reduced electron transport efficiency, and osmolyte-driven increases in structural biomass (Siddiqui, Al-Whaibi & Basalah, 2011; Munns, 2002).

PCA results reinforce these mechanistic distinctions. In juvenile plants, drought primarily influenced coordinated photochemical responses (F_0_–*F*_V_ clusters) and electron transport (PC2), consistent with osmotic limitation of PSII turnover (Ashraf, Nawazish & Athar, 2007; Kalaji et al., 2016). EC stress induced broader dispersion across components related to light absorption, electron flow, and yield, indicating complex reconfiguration of photosystem architecture and energy distribution under ionic stress (Kalaji et al., 2016; Munns, 2002). For mature plants, stronger clustering along PC1 under both stresses suggests more streamlined, uniform regulatory responses dominated by a few key photosynthetic control points (Siddiqui, Al-Whaibi & Basalah, 2011).

A key strength of the study is the integration of continuous-excitation fluorescence (JIP-test) and PAM fluorescence. JIP-test parameters quantified stress impacts on PSII’s potential efficiency and reaction-center structure (Strasser, Tsimilli-Michael & Srivastava, 2004; Kalaji et al., 2016; Kalaji et al., 2014), whereas PAM detected dynamic limitations in actual photochemistry, non-photochemical quenching, and energy partitioning under illuminated conditions (Kalaji et al., 2016; Maxwell & Johnson, 2000). Differences between potential and realized photosynthetic activity highlight stress-induced photoprotective regulation, such as enhanced NPQ, or damage that only manifests under light, revealing physiological bottlenecks that one method alone would not capture (Murchie & Lawson, 2013). Together, these complementary measurements enabled us to distinguish between osmotic and ionic constraints on photosynthesis and to identify developmental differences in how basil modulates photochemistry under drought and salinity conditions.

Our results clearly demonstrated that drought and high electrical conductivity (EC) stress impair photosynthetic performance through distinct physiological mechanisms, fully supporting the initial hypothesis. Drought acted primarily as an osmotic stress, limiting excitation energy capture and transfer within PSII and inducing selective disruptions in the early stages of electron transport. This pattern reflects a typical water-deficit response, where reduced turgor and impaired water balance restrict photochemistry without causing broad structural damage to the photosystems. In contrast, high-EC stress triggered a much broader spectrum of bioenergetic disturbances, characteristic of combined osmotic and ionic stress. Elevated salt concentration disrupted energetic antenna function, reduced reaction-center connectivity, impaired downstream electron-transport efficiency, and decreased key indicators of PSII integrity, such as PIabs and PItot. These stress-specific differences were also evident in the PAM fluorescence response: drought primarily increased photochemical limitations (reduced Yield and qP), whereas high EC strongly activated thermal dissipation and non-photochemical quenching mechanisms (increased qN and NPQ). The PCA further confirmed that the two stressors generated distinct physiological “signatures”, with drought mainly affecting electron-transport dynamics and high EC influencing antenna performance, energy distribution, and photoprotective regulation. Together, these findings show that basil plants deploy fundamentally different strategies to regulate and protect their photosystems under drought *versus* salt stress, which has important implications for optimizing stress management in hydroponic production systems.

## Conclusions

These results demonstrate that high–electrical-conductivity stress impairs the performance of basil plants more strongly than drought, highlighting salinity as a particularly restrictive abiotic factor. Nevertheless, basil mounts adaptive responses, namely, increased dry-matter accumulation and elevated chlorophyll content, that likely support its resilience. Both stressors elicited pronounced shifts in chlorophyll fluorescence parameters and biomass allocation, with distinct response patterns in juvenile *versus* mature plants. Juvenile plants displayed more pronounced alterations in electron-transport dynamics and photosystem function, reflecting their heightened sensitivity. In contrast, mature plants appeared to prioritize sustaining photosynthetic efficiency and limiting stress-induced damage.

This work underscores the complexity of basil’s physiological adaptations to abiotic stress and highlights plant developmental stage as a critical determinant of tolerance. These insights can guide the selection of more resilient cultivars and the refinement of irrigation and nutrient-management protocols. Further molecular and biochemical studies are recommended to validate the physiological mechanisms inferred from chlorophyll fluorescence responses.

##  Supplemental Information

10.7717/peerj.20728/supp-1 Supplemental Information 1 Raw data

## Additional Information and Declarations

### Data Availability

The following information was supplied regarding data availability: The raw measurements are available in the Supplemental File.
